# NEFM DNA methylation correlates with immune infiltration and survival in breast cancer

**DOI:** 10.1186/s13148-021-01096-4

**Published:** 2021-05-17

**Authors:** Dandan Li, Wenhao Zhao, Xinyu Zhang, Hanning Lv, Chunhong Li, Lichun Sun

**Affiliations:** 1 Department of Radiotherapy Oncology, Harbin Medical University Cancer Hospital, Harbin, China; 2 Department of Breast Medical Oncology, Harbin Medical University Cancer Hospital, No.150 Haping Road, Nangang District, Harbin, 150081 China

**Keywords:** Breast cancer, NEFM, DNA methylation, Lymphocytes, Tumor-infiltrating, Prognosis

## Abstract

**Background:**

This study aims to determine whether NEFM (neurofilament medium) DNA methylation correlates with immune infiltration and prognosis in breast cancer (BRCA) and to explore NEFM-connected immune gene signature.

**Methods:**

NEFM transcriptional expression was analyzed in BRCA and normal breast tissues using Oncomine and Tumor Immune Estimation Resource (TIMER) databases. The relationship between NEFM DNA methylation and NEFM transcriptional expression was investigated in TCGA. Potential influence of NEFM DNA methylation/expression on clinical outcome was evaluated using TCGA BRCA, The Human Protein Atlas and Kaplan–Meier plotter databases. Association of NEFM transcriptional expression/DNA methylation with cancer immune infiltration was investigated using TIMER and TISIDB databases.

**Results:**

High expression of NEFM correlated with better overall survival (OS) and recurrence-free survival (RFS) in TCGA BRCA and Kaplan–Meier plotter, whereas NEFM DNA methylation with worse OS in TCGA BRCA. NEFM transcriptional expression negatively correlated with DNA methylation. NEFM DNA methylation significantly negatively correlated with infiltrating levels of B, CD8^+^ T/CD4^+^ T cells, macrophages, neutrophils and dendritic cells in TIMER and TISIDB. NEFM expression positively correlated with macrophage infiltration in TIMER and TISIDB. After adjusted with tumor purity, NEFM expression weekly negatively correlated with infiltration level of B cells, whereas positively correlated with CD8^+^ T cell infiltration in TIMER gene modules. NEFM expression/DNA methylation correlated with diverse immune markers in TCGA and TISIDB.

**Conclusions:**

NEFM low-expression/DNA methylation correlates with poor prognosis. NEFM expression positively correlates with macrophage infiltration. NEFM DNA methylation strongly negatively correlates with immune infiltration in BRCA. Our study highlights novel potential functions of NEFM expression/DNA methylation in regulation of tumor immune microenvironment.

**Supplementary Information:**

The online version contains supplementary material available at 10.1186/s13148-021-01096-4.

## Introduction

Breast cancer (BRCA) is the most common malignancy among females worldwide. Clinical outcome has been improved over the past two decades with currently available modalities, including surgery, chemotherapy, endocrine therapy, radiotherapy, and targeted therapy, but BRCA treatment remains challenging because of high heterogeneity [1–3]. Immunotherapy is emerging as new therapeutics in BRCA. Several immunotherapeutic agents have been explored in various tumors, including adoptive cell therapies, vaccines, oncolytic viruses, and most notably immune check point blockade (ICB). Agents of ICB such as inhibitors of cytotoxic T-lymphocyte-associated antigen (CTLA-4), programmed cell death receptor1 (PD-1), and programmed cell death1 ligand1 (PD-L1) have been widely used in solid tumors, refractory cancers harboring microsatellite instability and classical Hodgkin lymphoma. Notably, anti-PD-L1 antibody atezolizumab in combination with nab-paclitaxel has been approved for the treatment of metastatic triple-negative breast cancer (TNBC) [1–4]. Expression of PD-L1 in infiltrating immune cells is required for response to atezolizumab plus nab-paclitaxel in IMpassion130 trial [5].

Tumor-infiltrating lymphocytes (TILs) comprise a mixture of cytotoxic T cells, helper T cells, B cells, macrophages, natural killer cells, and dendritic cells, which have been observed in many solid tumors, including BRCA. TILs may provide prognostic and predictive clues in BRCA and other cancers. To date, robust predictive biomarkers for immunotherapy have not been established in BRCA [1, 6]. TILs are more commonly observed at higher levels in TNBC and HER2-positive BRCA compared with estrogen receptor (ER)-positive and HER2-negative BRCA [1, 7, 8]. TILs may be associated with improved prognosis and better response rates to neoadjuvant therapy [7].

The NEFM (neurofilament medium), located on 8p21.2, encodes neurofilament medium polypeptide and assembles along with neurofilament heavy polypeptide (NEFH) and neurofilament light polypeptide (NEFL) into 10-nm filamentous structures, known as neurofilaments. Neurofilaments comprise axon skeleton functionally to maintain neuronal caliber and participate in intracellular transport to axons and dendrites. Neurofilaments have been implicated in biopathology of neurological diseases, including MDD (major depressive disorder) [8–11]. NEFM belongs to dopamine receptor-interacting protein (DRIP) gene family, which affects multi-aspects of dopamine receptor activity [12]. Besides, NEFM is associated with early response to antipsychotic medication [13]. Importantly, NEFM is involved in tumorigenesis/carcinogenesis [14–16]. NEFL and NEFM are located within 8p21, and LOH of this chromosome region has been described in several cancers including BRCA [17–19]. Additionally, NEFM is potentially involved in pancreatic cancer development and progression. Moreover, aberrant expression and methylation of neurofilament genes have been detected in ovarian cancer, esophageal squamous cell cancer, renal cell cancer, glioblastoma, neuroendocrine tumors, prostate cancer, uterine carcinosarcoma, Ewing sarcoma, hepatocellular cancer and BRCA [14–23]. DNA methylation-mediated silencing of neurofilament genes (NEFH, NEFM, NEFL) is a frequent event that contributes to the development and progression of BRCA [8].

In ovine amniotic epithelium (oAECs) isolated from late amnia, NEFM mRNA levels were significantly increased, while immunomodulatory effect of inhibiting lymphocyte proliferation was lost, and global DNA methylation was enhanced. Myelin oligodendrocyte glycoprotein induced incomplete tolerance of CD4 (+) T cells specific for myelin and neuronal self-antigen NEFM in mice [24, 25]. These studies suggest that NEFM is related to immune response. However, the relationship of NEFM with TILs in tumor progression or immunotherapy remains unclear.

In this study, association between NEFM expression and prognosis of BRCA was explored using TCGA (The Cancer Genome Atlas), The Human Protein Atlas, Oncomine and Kaplan–Meier plotter. In addition, association between NEFM DNA methylation and NEFM transcriptional expression was analyzed using BRCA samples in TCGA. Moreover, the relationship of NEFM transcriptional expression and NEFM DNA methylation with tumor-infiltrating immune cells was investigated in TCGA BRCA based on Tumor Immune Estimation Resource (TIMER) and TISIDB (tumor–immune system interactions).

## Results

### NEFM transcriptional expression levels in various cancers

**Fig. 1:**
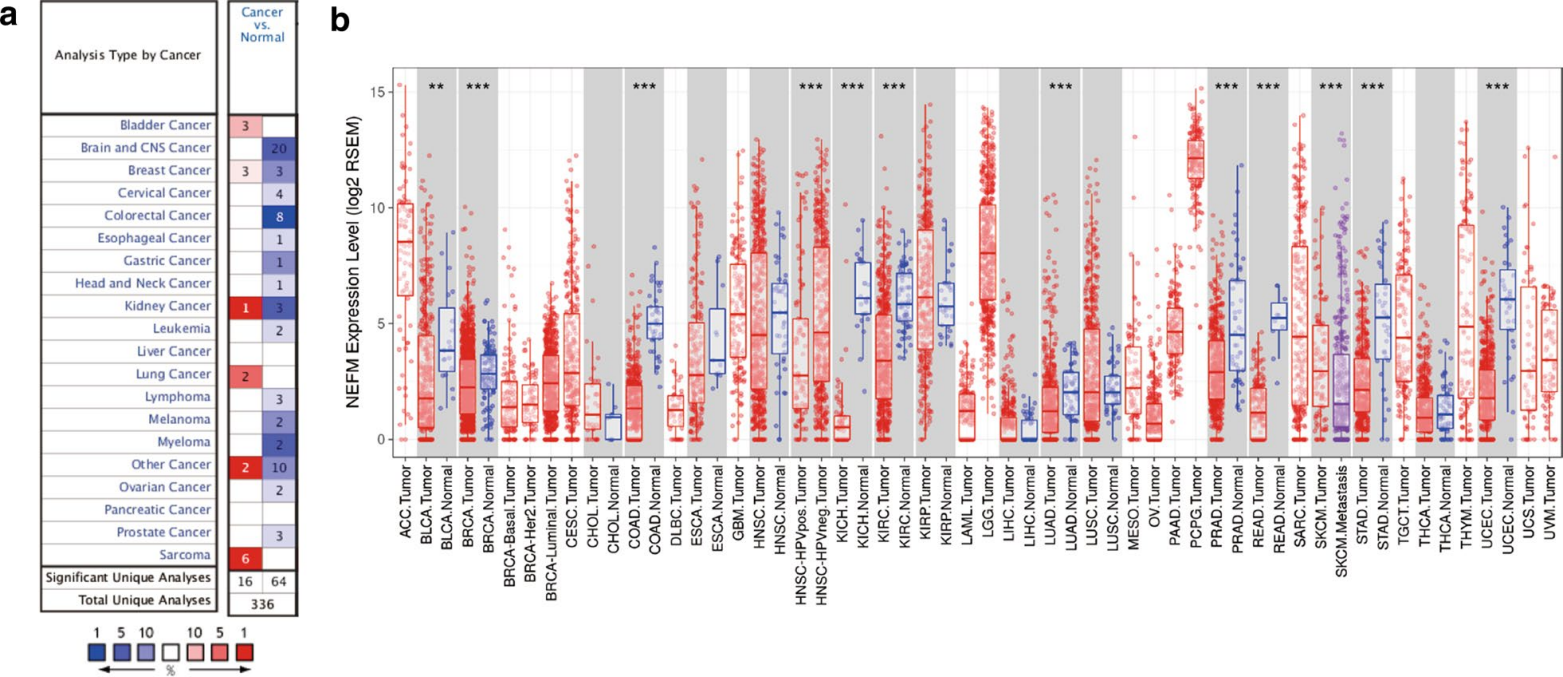
NEFM transcriptional expression levels in different types of human cancers. **a** Increased or decreased NEFM in different cancers compared with normal tissues in Oncomine database. **b** Human NEFM transcriptional expression levels in different tumor types from TCGA database as determined by TIMER. (*p < 0.05; **p < 0.01; ***p < 0.001)

Differential transcriptional expression of NEFM was profiled in tumor and adjacent non-malignant/normal tissues of multiple cancer types using Oncomine database. NEFM transcriptional expression was downregulated in most cancers, including brain and CNS, breast, colorectal, gastric, kidney, esophageal, ovarian, head and neck, cervical cancers, and lymphoma, while NEFM transcriptional expression was upregulated in bladder, breast, kidney, lung cancer, and sarcoma (Fig. 1a). To explore differential expression of NEFM between tumor and normal tissues, RNA-seq data derived from multiple malignancies in TCGA were examined by TIMER. NEFM expression was significantly lower in BLCA (bladder urothelial carcinoma), BRCA (breast invasive carcinoma), COAD (colon adenocarcinoma), HNSC (head and neck carcinoma), LUAD (lung adenocarcinoma), PRAD (prostate adenocarcinoma), READ (rectum adenocarcinoma), STAD (stomach adenocarcinoma), KICH (kidney chromophobe), KIRC (kidney renal clear cell carcinoma), and UCEC (uterine corpus endometrial carcinoma), compared with adjacent normal tissues. By contrast, NEFM expression was comparable between tumor and normal tissues in THCA (thyroid carcinoma), KIRP (kidney renal papillary cell carcinoma), CHOL (cholangiocarcinoma), ESCA (esophageal adenocarcinoma), LIHC (liver hepatocellular carcinoma), LUSC (lung squamous carcinoma), THCA (thyroid carcinoma) and LIHC (liver hepatocellular carcinoma) (Fig. 1b).

### Prognostic potential of NEFM in cancers

**Fig. 2:**
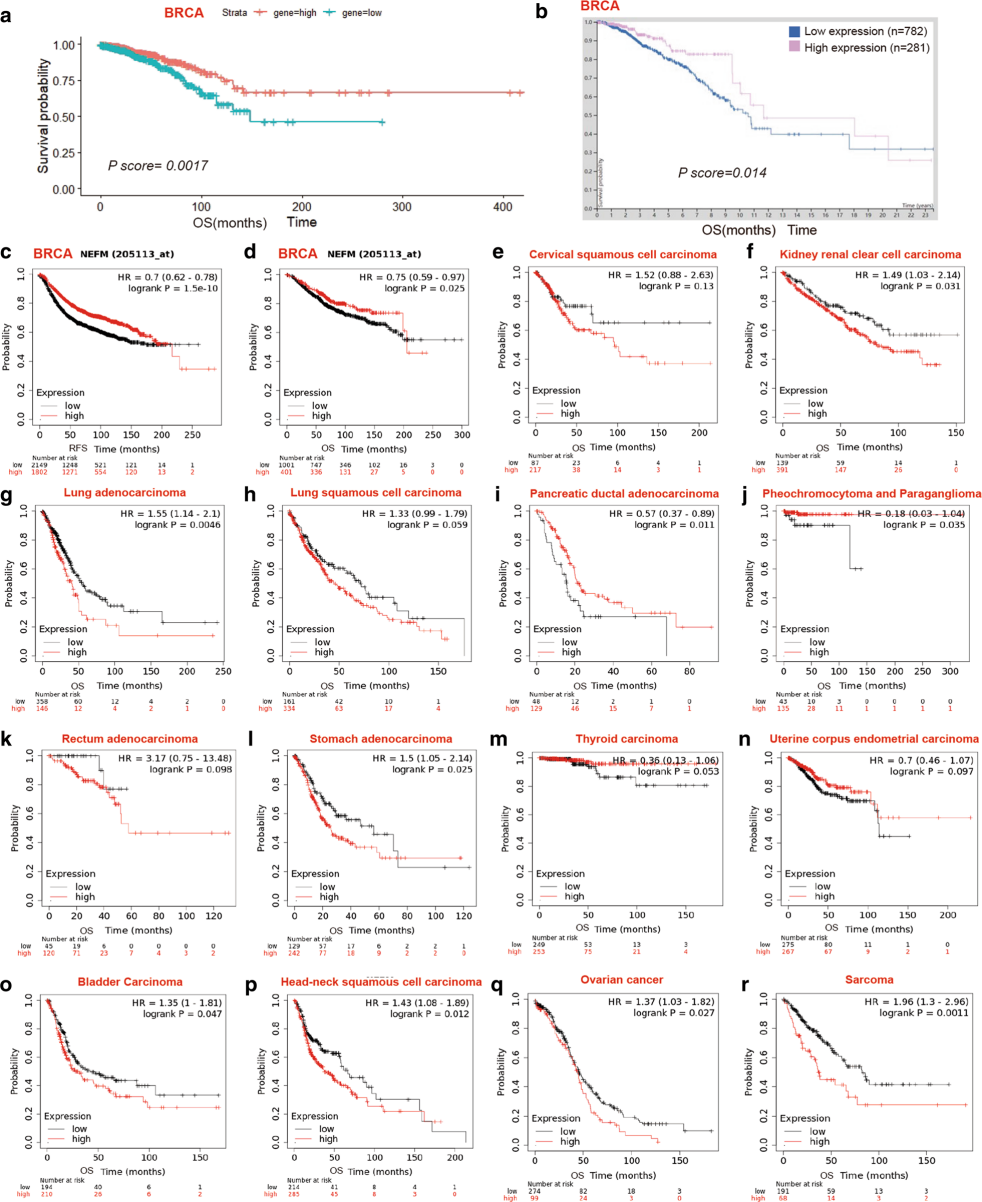
Kaplan–Meier survival curves of high versus low expression of NEFM in TCGA, Human Protein Atlas and Kaplan–Meier plotter databases. (OS: overall survival; NA: not applicable; RFS: recurrence-free survival). **a** OS curves of BRCA in TCGA. Low NEFM mRNA expression correlated with poor OS in TCGA_BRCA cohort (median OS: 149 vs. NA months, *p* = 0.0017). **b** OS curves of BRCA in Human Protein Atlas database. NEFM protein expression correlated with favorable OS (*p* = 0.0014). **c** RFS curves of BRCA in Kaplan–Meier plotter databases (median RFS: 37.8 vs. 69.2 months, *p* = 1.5e-10). **d** OS curves of BRCA in Kaplan–Meier plotter databases (median OS: 88.67 vs. 143 months *p* = 0.025). **e**–**r**. OS curves of pan_cancer in Kaplan–Meier plotter databases. **e** Cervical squamous cell carcinoma; **f** kidney renal clear cell carcinoma; **g** lung adenocarcinoma; **h** lung squamous cell carcinoma; **i** pancreatic ductal adenocarcinoma; **j** pheochromocytoma and paraganglioma; **k** rectum adenocarcinoma; **l** stomach adenocarcinoma; **m** thyroid carcinoma; **n** uterine corpus endometrial carcinoma; **o** bladder carcinoma; **p** head–neck squamous cell carcinoma; **q** ovarian cancer; **r** Sarcoma. High NEFM expression correlated with favorable OS of pancreatic ductal adenocarcinoma, pheochromocytoma and paraganglioma, whereas with poor OS of kidney renal clear cell carcinoma, lung adenocarcinoma, stomach adenocarcinoma, bladder carcinoma, head–neck squamous cell carcinoma, ovarian cancer and sarcoma in Kaplan–Meier plotter databases

Potential impact of NEFM expression on overall survival (OS) was evaluated in Pan-cancer RNA-seq in Kaplan–Meier plotter (Table 1, Fig. 2e–r.) Notably, a higher level of NEFM expression correlated with favorable OS of pancreatic ductal adenocarcinoma, pheochromocytoma and paraganglioma, whereas with poor OS of kidney renal clear cell carcinoma, lung adenocarcinoma, stomach adenocarcinoma, bladder carcinoma, head–neck squamous cell carcinoma, ovarian cancer and sarcoma as demonstrated in Kaplan–Meier plotter databases.

**Table 1:** Impact of NEFM on overall survival (OS) in Pan-cancer RNA-seq in Kaplan–Meier plotter

Cancers	No. of patients	MST (OS,Month)		HR	*p*
		NEFM high expression	NEFM low expression		
Kidney renal clear cell carcinoma	530	36.57	52.23	1.49	**0.031**
Lung adenocarcinoma	501	40.3	54.4	1.55	**0.0046**
Stomach adenocarcinoma	371	25.97	56.2	1.5	**0.025**
Bladder carcinoma	405	31.37	42.33	1.35	**0.047**
Head–neck squamous cell carcinoma	499	37.8	66.73	1.43	**0.012**
Ovarian cancer	373	43.8	45.47	1.37	**0.027**
Sarcoma	259	36.27	82.13	1.96	**0.0011**
Pancreatic ductal adenocarcinoma	177	22.03	15.57	0.57	**0.011**
Cervical squamous cell carcinoma	304	29.3	68.4	1.52	0.13
Lung squamous cell carcinoma	495	44.87	71.1	1.33	0.059
Rectum adenocarcinoma	502,	NA	NA	3.17	0.098
Thyroid carcinoma	502	NA	NA	0.36	0.053
Pheochromocytoma and paraganglioma	178	NA	NA	0.18	**0.035**
Uterine corpus endometrial carcinoma	543	103.73	51.6	0.7	0.097

Significant *p* value < 0.05 is in bold

Lower NEFM expression (n = 545; MST: 149 months) was associated with worse OS, compared to higher expression (n = 544; MST: NA, and *p* = 0.0017) (Fig. 2a) in females in (TCGA) BRCA cohort, while NEFM protein expression correlated with favorable OS (*p* = 0.0014) in Human Protein Atlas database (Fig. 2b). In univariable Cox proportional hazards regression models clinical stage (*p* < 0.001), NEFM expression (*p* = 0.002), menopause (*p* = 0.04), age (*p* < 0.001), ER (*p* = 0.007), PR (*p* = 0.013), HER2 (*p* = 0.021) were prognostic factors for OS. Furthermore, clinical stage (*p* < 0.001), age (*p* = 0.001), and NEFM expression (*p* = 0.031) remained as independent prognostic factors of OS in multivariable Cox proportional hazards regression model (Table 2). To further explore prognostic potential of NEFM in tumors, Kaplan–Meier plotter derived from Affymetrix microarrays was applied. In accordance with TCGA BRCA, higher NEFM expression correlated with better prognosis of BRCA (OS: MST: 143 vs. 88.67 months for high vs. low NEFM expression, n = 1402, HR = 0.75, 95% CI 0.59–0.97, *p* = 0.025; recurrence-free survival (RFS): MST: 69.2 vs. 37.8 months, n = 3951, HR = 0.7, 95% CI 0.62–0.78, *p* = 1.5e−10) (Fig. 2c, d).

**Table 2:** Univariable and multivariable Cox proportional hazards regression models of NEFM expression with clinicopathological features in TCGA BRCA cohort

Characteristics	Univariate			Multivariate		
	HR	CI95	*p*	HR	CI95	*p*
Subgroup	1.03	0.89–1.2	0.648			
Stage	1.8	1.38–2.35	** < 0.001**	1.83	1.34–2.51	** < 0.001**
NEFM	0.53	0.36–0.8	**0.002**	0.58	0.36–0.95	**0.031**
Age	1.03	1.02–1.05	** < 0.001**	1.03	1.01–1.05	**0.001**
ER	0.56	0.37–0.85	**0.007**	0.62	0.3–1.28	0.198
PR	0.6	0.4–0.9	**0.013**	0.67	0.33–1.37	0.271
HER2	1.75	1.09–2.8	**0.021**	1.61	0.96–2.69	0.071

HR: hazard ratio; CI 95: 95% confidence interval; subgroup: luminal A, luminal B, positive HER2, basal, normal; stage: I, II, III, IV; NEFM: low or high expression by median. Age, ER, PR, HER2 was divided into two groups according to the median, respectively. Significant *p* values < 0.05 are in bold

### Inverse correlation of NEFM DNA methylation with NEFM transcriptional expression

**Fig. 3:**
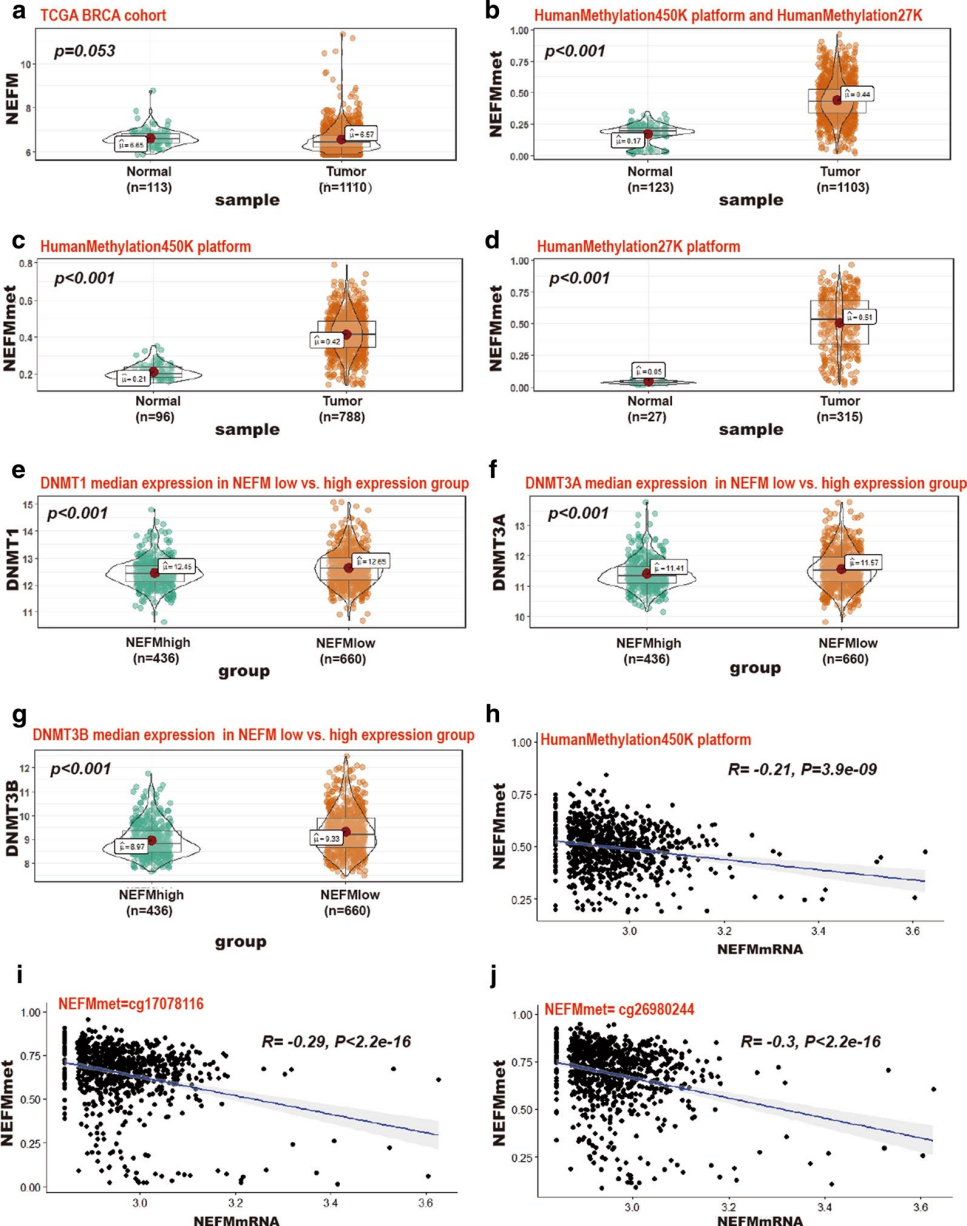
The correlation between NEFM transcriptional expression and DNA methylation of NEFM in TCGA BRCA cohort. **a** NEFM transcriptional expression was downregulated in BRCA compared with normal breast tissue. **b**–**d** NEFM DNA methylation was enhanced in BRCA compared with normal breast tissue (**b** NEFMmet = mean beta value of NEFM DNA methylation using 24 probe of HumanMethylation450K platform and 2 probe of HumanMethylation27K; **c** NEFMmet = mean beta value of NEFM DNA methylation of HumanMethylation450K platform; **d** NEFMmet = mean beta value of NEFM DNA methylation of HumanMethylation27K platform). **e**–**g** Significant higher DNA methyltransferase was connected with NEFM low expression. **e** DNMT1 median expression 12.65 vs. 12.45 in NEFM low vs. high-expression group, *p* < 0.001; **f** DNMT3A median expression 11.57 vs. 11.41 in NEFM low vs. high-expression group, *p* < 0.001; **g** DNMT1 median expression 9.33 vs. 8.97 in NEFM low- vs. high-expression group, *p* < 0.001). **h**–**j**. DNA methylation of NEFM inversely correlated with NEFM expression. **h** NEFMmet = log2 mean beta value of NEFM DNA methylation of 24 probe of HumanMethylation450K platform, HR = − 0.21, *p* = 3.9e−09; **i** NEFMmet = log2 beta value of cg17078116 probe, HR = − 0.29, *p* < 2.2e−16; **j** NEFMmet = log2 beta value of cg26980244 probe, HR = − 0.30, *p* < 2.2e−16

Genome-wide DNA methylation array and gene expression profiles of breast tissues from TCGA were explored to investigate the relationship of DNA methylation with transcriptional expression of NEFM. Methylation levels of NEFM were tested in Illumina Infinium HumanMethylation450 array and Illumina Infinium HumanMethylation27 array based on 24 and 2 Infinium probes, respectively, in 1103 tumors versus 123 normal breast tissues (788 tumors vs. 96 normal with HumanMethylation450 array; 315 tumors vs. 27 normal with HumanMethylation27 array). Comparing with normal tissues, higher levels of NEFM DNA methylation of NEFM were observed in tumors (Fig. 3b–d), while NEFM transcriptional expression was lower in tumor based on BRCA Illumina HiSeq RNA-Seq dataset including 1110 tumors versus 113 normal breast tissues (Fig. 3a). In addition, levels of 3 DNA methyltransferases were significantly different between NEFM high-expression group and low-expression group. Higher levels of DNMT1 (12.45 vs. 12.65), DNMT3A (11.41 vs. 11.57), DNMT3B (8.97 vs. 9.33) were observed in NEFM low-expression group (Fig. 3e–g, *p* < 0.001). Integrated analysis confirmed the inverse relationship of NEFM DNA methylation in Illumina Infinium HumanMethylation450 array with NEFM transcriptional expression in TCGA breast tumors (Fig. 3h–j). In TCGA BRCA HumanMethylation450K cohort, higher level of NEFM DNA methylation of NEFM was associated with poor OS (HR = 1.6 *p* = 0.035) (Table 3). Six loci of NEFM DNA methylation were significantly associated with OS in BRCA based on univariable Cox proportional hazards regression survival analysis (cg02761376, HR = 1.56, *p* = 0.045; cg07502389, HR = 1.76, *p* = 0.012; cg09234518, HR = 1.96, *p* = 0.003; cg18267374, HR = 1.75, *p* = 0.013; cg19677607, HR = 1.6, *p* = 0.038; cg26330518, HR = 1.56, *p* = 0.044). cg26330518 is located in promoter N_Shore, the other five loci are located in promoter CpG island region.

**Table 3:** Univariable Cox proportional hazards regression survival analyses of different NEFM DNA methylation loci in TCGA BRCA HumanMethylation450K platform

VarNames	UCSC_RefGene_Group	Relation_to_UCSC_CpG_Island	HR	CI95	*p*
NEFMmet			1.6	1.03–2.48	**0.036**
cg01583969	5'UTR;Body	S_Shore	1.47	0.95–2.28	0.086
cg02002551	5'UTR;Body	S_Shore	1.14	0.74–1.76	0.545
cg02106941	TSS1500;1stExon	Island	0.97	0.63–1.49	0.888
cg02761376	TSS1500;1stExon	Island	1.56	1.01–2.42	**0.045**
cg03012544	Body;Body	S_Shore	1.14	0.74–1.75	0.555
cg03169018	TSS200;Body	Island	1.2	0.78–1.84	0.412
cg04118306	TSS200;1stExon	Island	0.97	0.63–1.49	0.885
cg07502389	TSS200;TSS1500	Island	1.76	1.14–2.74	**0.012**
cg07552803	TSS1500;1stExon	Island	1.31	0.85–2.02	0.216
cg09234518	TSS200;TSS1500	Island	1.96	1.25–3.07	**0.003**
cg12026749	5'UTR;Body	S_Shore	1.24	0.8–1.9	0.335
cg13387869	3'UTR;3'UTR	S_Shelf	0.84	0.55–1.29	0.424
cg16459364	TSS200;TSS1500	Island	1.14	0.74–1.76	0.557
cg17078116	TSS200;1stExon	Island	1.11	0.72–1.7	0.647
cg18267374	TSS1500;5'UTR;1stExon	Island	1.75	1.13–2.72	**0.013**
cg18898125	TSS1500	N_Shore	1.22	0.79–1.87	0.368
cg19677607	TSS200;1stExon	Island	1.6	1.03–2.48	**0.038**
cg20585869	TSS200;1stExon	Island	0.78	0.5–1.2	0.261
cg22562942	TSS200;1stExon	Island	1.35	0.87–2.1	0.181
cg23290344	TSS1500;1stExon	Island	1.34	0.87–2.06	0.188
cg24705551	Body;Body	S_Shelf	1.12	0.73–1.72	0.596
cg26330518	TSS1500	N_Shore	1.56	1.01–2.41	**0.044**
cg26980244	5'UTR;1stExon;Body	Island	1.42	0.92–2.19	0.116
cg27475652	Body;Body	S_Shelf	1.04	0.67–1.6	0.867

HR: hazard ratio; CI 95: 95% confidence interval. Significant *p* Values < 0.05 are in bold. NEFMmet: mean beta value of all DNA methylation loci of NEFM. Seven DNA methylation loci of NEFM including NEFMmet were connected with survival

### The genes and pathways connected with NEFM transcriptional expression/DNA methylation

**Fig. 4:**
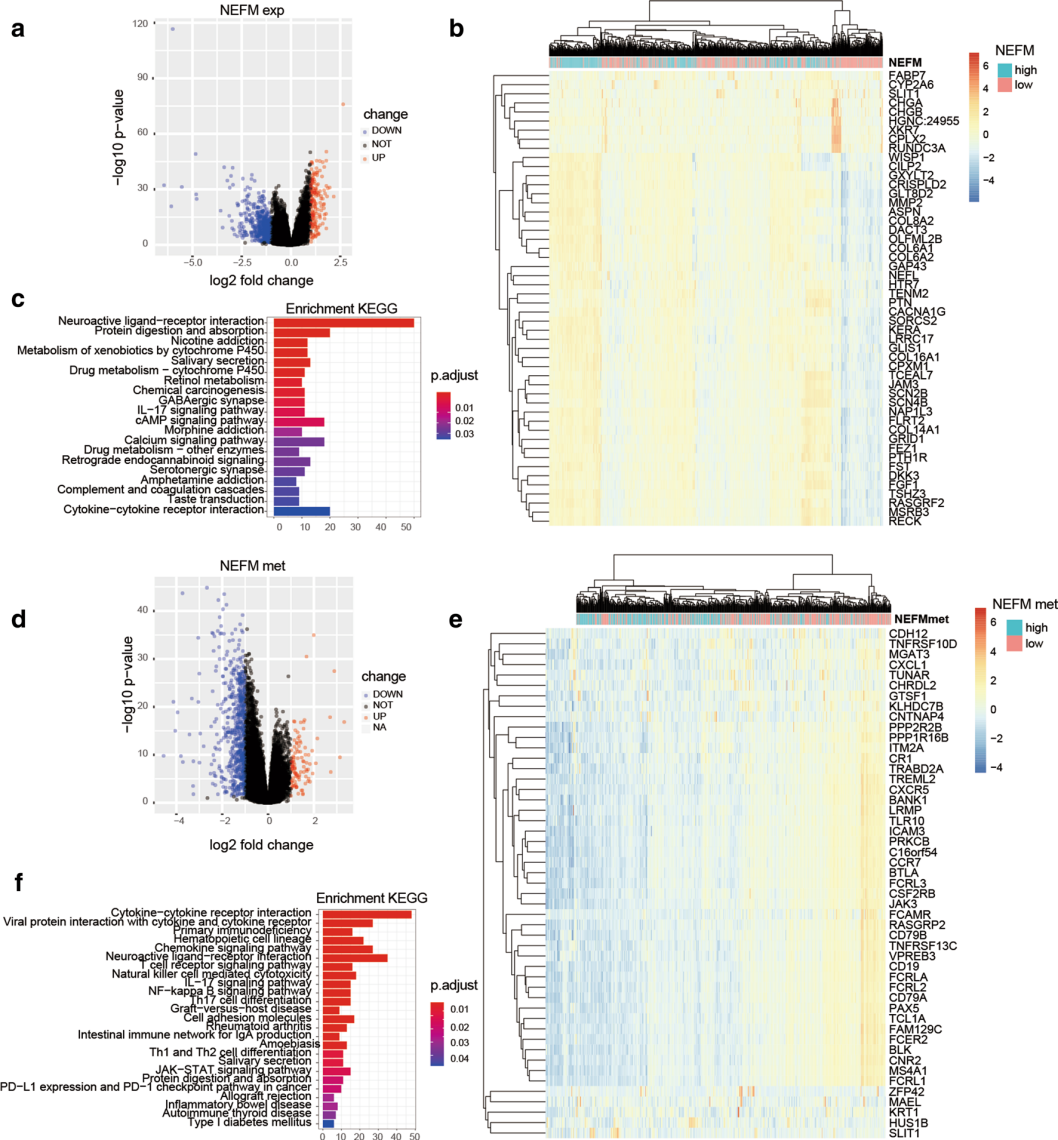
Genes and pathways connected with NEFM transcriptional expression/DNA methylation in TCGA BRCA cohort. **a** Volcano plot of differentially expressed gene profiles between NEFM high-expression group and NEFM low-expression group (absolute log2 (fold change) > 1, adjp < 0.05). **b** Expression heatmap of the top 50 NEFM-associated genes. The top curve described NEFM's expression distribution of 1096 BRCA samples. **c** Bar plot of the NEFM expression_related signaling pathways derived from KEGG Eenrichment analysis. **d** Volcano plot of differentially expressed gene profiles between NEFM high DNA methylation group and NEFM low DNA methylation group (absolute log2 (fold change) > 1, adjp < 0.05). **e** Expression heatmap of the top 50 NEFM DNA methylation-associated genes. The top curve described NEFM methylation distribution of 691 BRCA samples. **f** Bar plot of NEFM methylation-related signaling pathways derived from KEGG enrich analysis

Differentially expressed genes associated with NEFM expression or NEFM DNA methylation were profiled through comparison between NEFM/NEFMmet high and low groups in TCGA BRCA cohort. Totally, 164 up-regulated and 546 down-regulated genes were significantly associated with NEFM expression, while 103 up-regulated and 641 down-regulated genes were significantly associated with NEFM DNA methylation (with absolute value of log2foldchange > 1, and adjust *p* value < 0.05; Fig. 4a, d; Additional files 1, 2: Tables S1–S2). The top 50 differentially expressed genes were presented as expression heatmaps (Fig. 4b, e). Critical signal transduction pathways involved in NEFM expression included neuroactive ligand-receptor interaction, protein digestion and absorption, chemical carcinogenesis, cAMP signaling pathway, IL-17 signaling pathway, and cytokine–cytokine receptor interaction by KEGG enrichment analysis (Fig. 4c). Cytokine–cytokine receptor interaction, viral protein interaction with cytokine and cytokine receptor, primary immunodeficiency, hematopoietic cell lineage, chemokine signaling pathway, neuroactive ligand–receptor interaction, T cell receptor signaling pathway, natural killer cell-mediated cytotoxicity, IL-17 signaling pathway, and NF-kappa B signaling pathway were the top 10 pathways closely associated with NEFM DNA methylation based on KEGG enrichment analysis. Notably, some pathways involved in immune response such as Th17 cell differentiation, graft-versus-host disease, intestinal immune network for IgA production, Th1 and Th2 cell differentiation, as well as PD-L1 expression and PD-1 checkpoint pathway in cancer, were significantly associated with NEFM methylation (Fig. 4f).

### Correlation of NEFM transcription/DNA methylation with immune infiltration in breast cancer

**Fig. 5:**
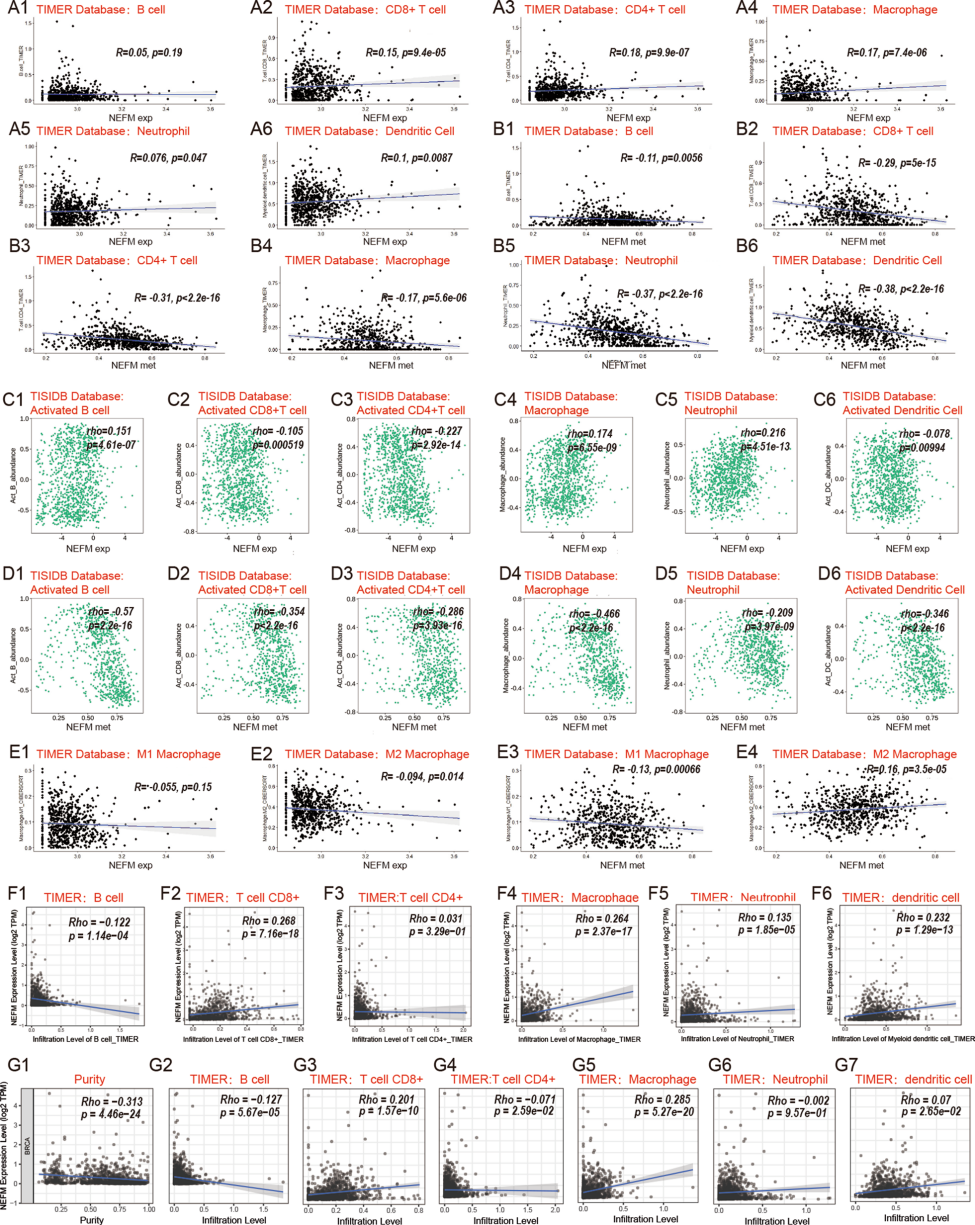
Correlation of NEFM transcriptional expression/DNA methylation with immune infiltration levels in BRCA. **a** NEFM transcriptional expression weakly positively correlated to infiltration levels of CD8 + T cells, CD4 + T cells and macrophages in BRCA in TIMER database by correlation analysis. NEFM expression showed very weak positive association with infiltration levels of neutrophils and dendritic cells in TCGA BRCA in TIMER database (n = 703). **b** DNA methylation of NEFM had significant negative association with infiltration levels of B cells, CD8 + T cells, CD4 + T cells, macrophages, neutrophils, and dendritic cells in TIMER database by correlation analysis (n = 703). **c** NEFM expression had weak positive association with infiltration levels of activated B cells, macrophages and moderate positive association with infiltration levels of neutrophils, whereas negative association with infiltration levels of activated CD8 + T cells, activated CD4 + T cells and activated dendritic cells in BRCA in TISIDB database (n = 1100). **d** DNA methylation of NEFM significantly negatively correlated with infiltration levels of activated B cells, activated CD8 + T cells, activated CD4 + T cells, macrophages, neutrophils, and activated dendritic cells in TISIDB database (n = 785). **e** NEFM expression weakly negatively correlated with infiltration levels of M2 macrophage; DNA methylation of NEFM negatively correlated with infiltration levels of M1 macrophage whereas positively correlated with infiltration levels of M2 macrophage in TIMER database (n = 703). **f** NEFM transcriptional expression positively correlated with infiltration levels of CD8 + T cells, macrophages, neutrophils, dendritic cells and negatively correlated with infiltration level of B cells in TIMER gene modules (n = 1100). **e** After adjusted by tumor purity, NEFM expression weekly negatively correlated with infiltration level of B cell and positively correlated with infiltration levels of macrophages and CD8 + T cells in TIMER gene modules (n = 1100)

Relationship of NEFM transcriptional expression/DNA methylation with immune infiltration in breast cancer was assessed using correlation analysis and TISIDB databases. NEFM transcriptional expression was weakly (R < 2) to moderately (2 < R < 3) positively associated with infiltration levels of macrophages and neutrophils using correlation analysis and TISIDB database (Fig. 5a, c). NEFM transcriptional expression was weakly positively associated with infiltration levels of CD8 + T cells, CD4 + T cells by correlation analysis, whereas weakly to moderately negatively associated with infiltration levels of activated CD8 + T cells, activated CD4 + T cells in TISIDB database (Fig. 5a, c). Since the different results from correlation analysis and TISIDB databases, TIMER gene modules were applied to evaluate the relationship of NEFM transcriptional expression with immune infiltration in breast cancer. In TIMER gene modules, NEFM transcriptional expression positively correlated with infiltration levels of CD8^+^ T cell, macrophage, neutrophil, and dendritic cell, and negatively correlated with infiltration level of B cell and tumor purity, whereas not with infiltration level of CD4^+^ T cell. After adjusted with tumor purity, NEFM expression weekly negatively correlated with infiltration level of B cell and positively correlated with macrophage and CD8^+^ T cell. NEFM DNA methylation was moderately to strongly (R > 3) negatively associated with infiltration levels of B cells, CD8 + T cells, CD4 + T cells, macrophages, neutrophils, and dendritic cells using correlation analysis and TISIDB database (n = 785) (Fig. 5b, d). Interestingly, NEFM transcriptional expression weakly negatively correlated to infiltration levels of M2 macrophage, while NEFM DNA methylation weakly negatively correlated to infiltration levels of M1 macrophage and positively correlated to infiltration levels of M2 macrophage with correlation analysis (Fig. 5e). Collectively, NEFM expression positively correlated with macrophage infiltration in TIMER and TISIDB; after adjusted with tumor purity, NEFM expression also weekly negatively correlated with infiltration level of B cell and positively correlated with CD8^+^ T cell in TIMER gene modules. However, NEFM DNA methylation was significantly negatively associated with immune infiltration in breast cancer. NEFM expression/DNA methylation might play a specific role in immune infiltration in BRCA.

### Correlation of NEFM transcriptional expression/DNA methylation with immune markers

**Fig. 6:**
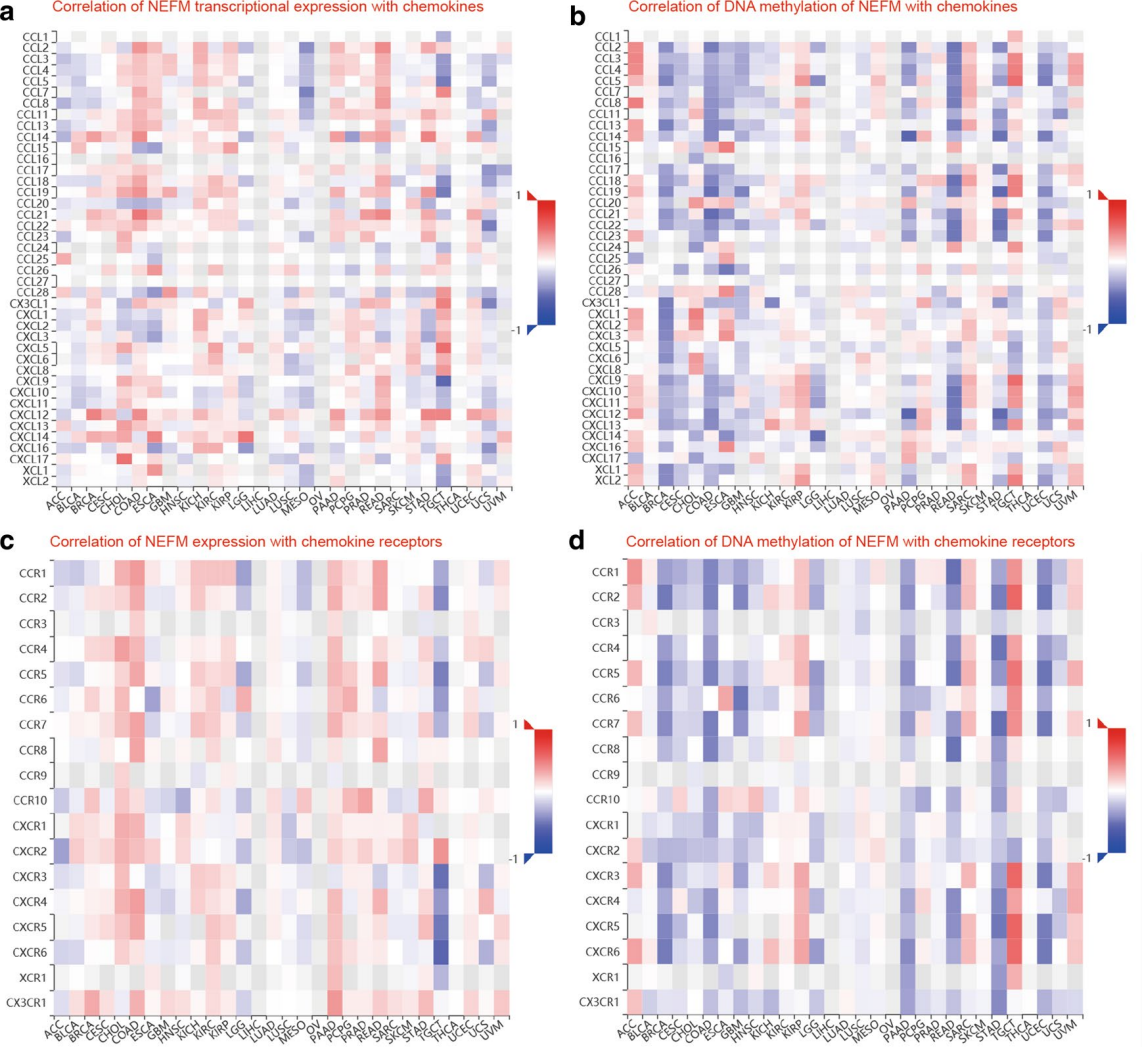
The correlation of NEFM transcriptional expression/DNA methylation with chemokines and receptors in TISIDB database. **a** Correlation of NEFM transcriptional expression with chemokines; **b** correlation of DNA methylation of NEFM with chemokines; **c** correlation of NEFM expression with chemokine receptors; **d** correlation of DNA methylation of NEFM with chemokine receptors

Relationship of NEFM transcriptional expression/DNA methylation with immune markers was evaluated using TISIDB and TCGA databases. NEFM transcriptional expression weakly to moderately correlated with ADORA2A, CSF1R, IDO1, KDR, LAG3, TGFBR1, VTCN1, C10orf54, CD276, CD40, CD70, ENTPD1, NT5E, PVR, TMEM173, TNFRSF13B, TNFRSF17, TNFSF4 (1 < R < 3) and strongly correlated with CXCL12 and TGFB1 (R > 3). Except positive relation with PVRL2 but no relation with CD276, RAET1E, TNFRSF14, TNFRSF18, or TNFSF13, NEFM DNA methylation significantly negatively correlated with almost all immunomodulators collected from Charoentong's study (Table 4). NEFM transcriptional expression was weakly associated with major histocompatibility complex (MHC)-related molecules B2M, HLA-DPB1. Except TAPBP, NEFM DNA methylation was significantly negatively associated with all MHC-related molecules listed in TISIDB database (Table 5). NEFM transcriptional expression significantly positively correlated with CCL14, CCL21, CXCL12, CXCL14, CCL28, CCR10 and CX3CR1 and negatively correlated with CCL7, CCL8, CCL18, while NEFM DNA methylation was significantly negatively associated with most chemokines and receptors listed in TISIDB (Fig. 6).

**Table 4:** Correlation of NEFM transcriptional expression/DNA methylation with immunomodulators based on TISIDB and TCGA database

Immunomodulators	NEFM expression		NEFM DNA methylation	
	TISIDB rho, n=1100 (TCGA R, n = 808)	*p*	TISIDB rho, n=785 (TCGA R, n = 808)	*P*
ADORA2A	0.137 (0.15)	**5.07e−6 (2.5e−05)**	− 0.095 (− 0.004)	**0.00751 **(0.26)
BTLA	0.032 (0.041)	0.296 (0.240)	− 0.477 (− 0.35)	**2.2e−16**
CD160	− 0.019 (0.07)	0.539 (**0.046**)	− 0.157 (− 0.15)	**9.61e−06 (1.5e−05)**
CD244	0.06 (0.076)	**0.0467 (0.032)**	− 0.525 (− 0.35)	** < 2.2e−16**
CD274(PD-L1)	− 0.006 (0.038)	0.854 (0.28)	− 0.304 (− 0.28)	**4.23e−18 (2.3e−15)**
CD96	0.037 (0.042)	0.226 (0.23)	− 0.479 (− 0.34)	** < 2.2e−16**
CSF1R	0.132 (0.11)	**0.22e−05 (0.0027)**	− 0.44 (− 0.34)	** < 2.2e−16**
CTLA4	− 0.028 (0.00073)	0.357 (0.98)	− 0.432 (0.3)	** < 2.2e−16**
HAVCR2	0.008 (− 0.0079)	0.789 (0.82)	− 0.298 (− 0.21)	**2.16e−17 (1.8e−09)**
IDO1	− 0.112 (− 0.1)	**0.000203 (0.0036)**	− 0.384 (− 0.26)	** < 2.2e−16 (1.2e−13)**
IL10	− 0.003 (− 0.025)	0.921 (0.48)	− 0.388 (− 0.29)	** < 2.2e−16**
IL10RB	− 0.08 (− 0.036)	**0.00821 **(0.31)	− 0.262 (− 0.2)	**1.08e−13 (1.3e−08)**
KDR(VEGFR)	0.127 (0.11)	**2.51e−05 (0.0017)**	− 0.117 (− 0.15)	**0.000984 (2.9e−05)**
LAG3	− 0.171 (− 0.14)	**1.11e−08 (4.5e−05)**	− 0.314 (− 0.2)	**2.38e−19 (6.9e−09)**
LGALS9	− 0.051 (− 0.059)	0.0912 (0.093)	− 0.293 (− 0.17)	**9.15e−17 (1.2e−06)**
PDCD1	0.007 (0.023)	0.816 (0.51)	− 0.43 (− 0.29)	** < 2.2e−16**
PDCD1LG2	0.066 (0.076)	**0.0281 (0.031)**	− 0.476 (− 0.34)	** < 2.2e−16**
PVRL2(NECTIN2)	0.027 (0.045)	0.378 (0.2)	0.32 (0.14)	**2.9e−20 (9.9e−05)**
TGFB1	0.32 (0.3)	** < 2.2e−16**	− 0.28 (− 0.2)3)	**1.91e−15 (3.6e−11)**
TGFBR1	0.214 (0.21)	**9.26e−13 (2.7e−09)**	− 0.178 (− 0.13)	**5.46e−07 (0.00014)**
TGFBR2	(0.35)	** < 2.2e−16**	(− 0.33)	** < 2.2e−16**
TIGIT	− 0.02 (− 0.0034)	0.509 (0.92)	− 0.446 (− 0.31)	** < 2.2e−16**
VTCN1	0.207 (0.21)	**4.34e−12 (2.4e−09)**	− 0.175 (− 0.07)	**8.63e−07 (0.048)**
C10orf54(VSIR, VISTA)	0.205 (0.18)	**7.97e−12 (2.3e−07)**	− 0.552 (− 0.39)	** < 2.2e−16**
CD27(TNFRSF7)	0.097 ( (0.11)	**0.00121 (0.0013)**	− 0.452 (− 0.31)	** < 2.2e−16**
CD276	0.168 (0.19)	**2.12e−08 (9.7e−08)**	− 0.038 (− 0.065)	0.284 (0.068)
CD28	0.042 (0.038)	0.16 (0.28)	− 0.458 (− 0.33)	** < 2.2e−16**
CD40	0.137 (0.15)	**5.1e−06 (2.2e−05)**	− 0.553 (− 0.38)	** < 2.2e−16**
CD40LG	0.088 (0.093)	**0.00346 (0.0081)**	− 0.457 (0.32)	** < 2.2e−16**
CD48	0.062 (0.062)	**0.0391 **(0.079)	− 0.458 (− 0.31)	** < 2.2e−16**
CD70	0.108 (0.11)	**0.000345 (0.0017)**	− 0.312 (− 0.17)	**3.39e−19 (1.4e−06)**
CD80	− 0.069	**0.0228**	− 0.259 (− 0.18)	**1.95e−13 (2.8e−07)**
CD86	− 0.028 (− 0.056)	0.357 (0.11)	− 0.375	** < 2.2e−16**
CXCL12	0.467 (0.43)	** < 2.2e−16**	− 0.32 (− 0.2)	**3.31e−20 (1.5e−08)**
CXCR4	0.053 (0.087)	0.0763(**0.013)**	− 0.313 (− 0.17)	**3.11e−19 (8.2e−07)**
ENTPD1(CD39)	0.267 (0.3)	**2.85e−19 (< 2.2e−16)**	− 0.271 (− 0.23)	**1.46e−14 (5.5e−11)**
ICOS	− 0.064 (− 0.044)	**0.0341 **(0.21)	− 0.42 (− 0.29)	** < 2.2e−16**
ICOSLG	0.045 (0.0042)	0.15 (0.91)	− 0.193 (− 0.027)	**5.69e−08 **(0.44)
IL2RA	− 0.033 (− 0.022)	0.276 (0.53)	− 0.454 (− 0.33)	** < 2.2e−16**
IL6	0.091 (0.087)	**0.00266 (0.014)**	− 0.425 (− 0.3)	** < 2.2e−16**
IL6R	− 0.012 (− 0.015)	0.7 (0.67)	− 0.341 (− 0.29)	** < 2.2e−16**
KLRC1	0.004 (0.017)	0.907 (0.64)	− 0.386 (− 0.28)	** < 2.2e−16 (2.7e−16)**
KLRK1	0.041 (0.075)	0.171 (**0.033**)	− 0.456 (− 0.28)	** < 2.2e−16 (4.3e−16)**
LTA	− 0.033 (− 0.015)	0.273 (0.66)	− 0.413 (− 0.28)	** < 2.2e−16 (1e−15)**
MICB	0.003 (− 0.0094)	0.93 (0.79)	− 0.047 (− 0.16)	0.186(**3e−06**)
NT5E(CD73)	0.224 (0.2)	**6.85e−14 (1e−08)**	− 0.306 (− 0.28)	**2.26e−18 (5.6e−16)**
PVR	− 0.204 (− 0.17)	**1.05e−11 (8.9e−07)**	− 0.134 (− 0.11)	**0.00017 (0.0019)**
RAET1E	0.094 (0.12)	**0.00182 (0.00065)**	− 0.073 (− 0.016)	**0.0411 **(0.65)
TMEM173(STING)	0.231 (0.2)	**1.14e−14 (6.5e−09)**	− 0.263 (− 0.19)	**9.54e−14 (1.1e−07)**
TNFRSF13B	0.202 (0.21)	**1.39e−11 (3.8e−09)**	− 0.419 (− 0.31)	** < 2.2e−16**
TNFRSF13C	0.068 (0.027)	**0.0237 **(0.44)	− 0.346 (− 0.26)	** < 2.2e−16 (4.6e−14)**
TNFRSF14	0.048 (0.055)	0.109 (0.12)	− 0.074 (0.044)	**0.0377 **(0.21)
TNFRSF17	0.113 (0.13)	**0.000181(0.00037)**	− 0.396 (− 0.27)	** < 2.2e−16 (3.9e−15)**
TNFRSF18	− 0.029 (− 0.04)	0.335 (0.25)	0.064 (− 0.021)	0.0727 (**0.00037**)
TNFRSF25	0.014 (0.078)	0.646 (**0.028**)	− 0.257 (− 0.14)	**3.01e−13 (4.3e−05)**
TNFRSF4	0.082 (0.082)	**0.00664 (0.019)**	− 0.281 (− 0.18)	**1.51e−15 (2.9e−07)**
TNFRSF8	0.086 (0.085)	**0.00424 (0.015)**	− 0.529 (− 0.37)	** < 2.2e−16**
TNFRSF9	0.03 (0.036)	0.319 (0.3)	− 0.443 (− 0.36)	** < 2.2e−16**
TNFSF13	− 0.015 (0.028)	0.617 (0.42)	0.047 (0.075)	0.189 (**0.035**)
TNFSF13B	− 0.015 (− 0.025)	0.623 (0.48)	− 0.343 (− 0.25)	** < 2.2e−16 (1.3e−12)**
TNFSF14	0.082 (0.084)	**0.00622 (0.017)**	− 0.45 (− 0.32)	** < 2.2e−16**
TNFSF15	0.095 (0.085)	**0.00153 (0.015)**	− 0.201 (− 0.12)	**1.36e−08 (0.00091)**
TNFSF4	0.149 (0.17)	**6.77e−07 (2.1e−06)**	− 0.081 (− 0.096)	**0.0228 (0.0064)**
TNFSF9	0.024 (0.086)	**0.00165 (0.015)**	− 0.256 (− 0.11)	**3.82e−13 (0.0012)**
ULBP1 (NKG2D)	− 0.029 (0.0054)	0.333 (0.88)	− 0.159 (− 0.015)	**7.37e−06 **(0.68)

Significant *p* value < 0.05 is in bold

**Table 5:** Correlation of NEFM transcriptional expression/DNA methylation with MHC molecules based on TISIDB database

MHC molecules	NEFM expression		NEFM DNA methylation	
	TISIDB rho, n = 1100	*p*	TISIDB rho, n = 785	*p*
B2M	− 0.11	**0.00025**	− 0.287	**2.85e−16**
HLA-A	− 0.093	**0.00205**	− 0.3	**1.23e−17**
HLA-B	− 0.089	**0.00388**	− 0.315	**1.58e−19**
HLA-C	− 0.077	**0.0107**	− 0.212	**2.35e−09**
HLA-DMA	0.058	0.0561	− 0.405	** < 2.2e−16**
HLA-DMB	0.03	0.313	− 0.414	** < 2.2e−16**
HLA-DOA	0.156	**2.16e−07**	− 0.458	** < 2.2e−16**
HLA-DOB	0.034	0.266	− 0.503	** < 2.2e−16**
HLA-DPA1	0.084	**0.00511**	− 0.407	** < 2.2e−16**
HLA-DPB1	0.153	**3.46e−07**	− 0.435	** < 2.2e−16**
HLA-DQA1	0.061	**0.0446**	− 0.398	** < 2.2e−16**
HLA-DQA2	0.057	0.058	− 0.294	**5.78e−14**
HLA-DQB1	0.081	**0.00747**	− 0.352	** < 2.2e−16**
HLA-DRA	0.061	**0.0447**	− 0.434	** < 2.2e−16**
HLA-DRB1	0.089	**0.00329**	− 0.39	** < 2.2e−16**
HLA-E	0.05	0.0991	− 0.484	** < 2.2e−16**
HLA-F	− 0.028	0.358	− 0.377	** < 2.2e−16**
HLA-G	− 0.04	0.189	− 0.231	**6.32e−11**
TAP1	− 0.204	**8.63e−12**	− 0.267	**3.39e−14**
TAP2	− 0.176	**4.89e−09**	− 0.381	** < 2.2e−16**
TAPBP	− 0.143	**1.91e−06**	− 0.057	0.109

Significant *p* value < 0.05 is in bold

## Discussion

In this study, NEFM transcriptional expression was downregulated and negatively correlated with DNA methylation in breast cancer. Enhanced DNA methylation on six loci within NEFM located on promoter CpG island or shore was associated with poor survival. Besides, NEFM transcriptional expression correlated with better prognosis and correlated with increased macrophage; after normalized with tumor purity, NEFM expression correlated with increased CD8^+^ T cell, whereas decreased B cell infiltration in BRCA. NEFM DNA methylation correlated with decreased infiltration levels of B cells, CD8 + T cells, CD4 + T cells, macrophages, neutrophils, and dendritic cells and diverse immune markers. Therefore, our study provides new evidence to support a role of NEFM transcriptional expression/NEFM DNA methylation in BRCA.

NEFM polypeptide is one of the four subunits comprising neurofilaments, the most abundant intermediate filaments in nervous system. In addition, NEFM, NEFL and NEFH act as onco-suppressors for affecting cell proliferation and correlate with worse prognosis [8, 31, 32]. It was reported that methylation-mediated inactivation of NEFH, NEFL or NEFM was common in primary breast tumors compared to normal breast tissues and correlated with clinical features of disease progression. DNA methylation-mediated inactivation of NEFH, NEFL and NEFM also occur in other types of cancer originated from pancreas, gastric and colon [8]. Consistently, we demonstrated that NEFM transcriptional expression was downregulated in most cancers including breast, colorectal, gastric, kidney, head and neck, compared with normal tissues in Oncomine and TIMER databases. NEFM DNA methylation negatively correlated with survival and NEFM transcriptional expression in BRCA. In our study, DNA methyltransferases (DNMT1, DNMT3A and DNMT3B) were highly expressed in NEFM low-expression group in TCGA BRCA samples, suggesting that DNMT1, DNMT3A and DNMT3B might contribute to NEFM silencing in BRCA. Emerging evidence indicates that promoter methylation is associated with gene silencing, development, progression and chemotherapy sensitivity of BRCA [2, 33, 34]. Therefore, the identification of novel tumor-suppressive genes targeted by promoter methylation can reveal tumor-suppressive pathways in breast carcinogenesis and explore alternative approaches for diagnostic and therapeutic evaluation. We investigated the relationship between 24 loci within NEFM gene and prognosis in BRCA and identified enhanced NEFM DNA methylation of six out of 16 loci located on promoter CpG island related to poor survival.

Elevated levels of anti-NEFM antibodies were detected in various neurological diseases, including autoimmune diseases, non-immune-mediated conditions, and even in individuals being considered normal or with disorders unrelated to intrathecal space, such as multiple sclerosis, schizophrenia, spondylogenic headache or neurastenia. Therefore, anti-NEFM antibodies may be regarded as natural circulating auto-antibodies [35, 36]. Poly-specific T cells targeting distinct self-antigens have been identified in healthy individuals as well as in the context of autoimmunity. T cell recognizes NEFM protein, with implications for aggravation and perpetuation of central nervous system autoimmunity [37]. However, whether NEFM is involved in regulating antitumor immunity with clinical significance in breast cancer remains unknown. In this study, positive association of NEFM expression with infiltration level of macrophage was replicated by correlation analysis, TIMER2.0 gene module and TISIDB; however, relationship of NEFM expression with infiltration levels of other TILs varied, possibly due to different numbers of available TCGA samples used for batch correction and differences in calculation methods. TIMER gene module (version 2.0) provides abundance of immune infiltration estimated by multiple immune deconvolution methods and adjusted by tumor purity, which is a major confounding factor in this analysis, and thus, the results are more accurate, with more reliable biological significance. Relationship of NEFM transcriptional expression with immunomodulators has implicated its involvement in regulating tumor immunology in BRCA. Firstly, macrophage markers IL6, CSF1R, CXCL12 were weak-to-strong positively associated with NEFM expression, which could reveal a potential role of NEFM transcriptional expression in regulating polarization of tumor-associated macrophage (TAM). In addition, NEFM transcriptional expression was positively associated with levels of T-cell exhaustion markers, specifically ADORA2A, VISTA and CCR4 [38, 39]. Moreover, NEFM was positively associated with ectonucleotidases CD39 and CD73, novel checkpoint inhibitors that interfere with anti-tumor immune responses [40]. NEFM negatively correlated with PVR (CD155), an immune checkpoint on tumor cells and interacting with CD96, CD226, and TIGIT (T cell immune receptor with immunoglobulin and ITIM domains) on TILs to modulate immune function in tumor microenvironment [41]. In addition, NEFM significantly positively correlated with TGFB1, TGFBR1. Depending on the presence of other secreted factors and cell surface co-receptors, TGFB can either suppress adaptive immune responses (through induction and stabilization of Tregs and directly suppressing Th1 cell, Th2 cell and CD8 + T cell) or enhance adaptive responses (through induction of Th17 cell, Th9 cell and CD4 + CTL-like effector cell) [42]. The relationship of NEFM with TILs in BRCA may partially rely on chemokines and chemokine receptors. More and more studies have shown that chemokines and chemokine receptors are closely related to the immunity of breast cancer. NEFM transcriptional expression was negatively associated with CCL7, CCL8 and CCL18, which would recruit monocytes to differentiate into tumor-associated macrophages (TAMs) at the tumor site, indicating that NEFM may cause decreased M2 macrophage infiltration [43]. Furthermore, NEFM transcriptional expression was positively associated with CCL14, CCL21, CXCL12, CXCL14, CCL28, CCR10 and CX3CR1. Notably, CX3CR1 promotes macrophage recruitment during mammary tumor formation. Macrophages are attracted to tumor sites expressing chemotactic factors such as CCL7, CCL8 and CXCL12 [43, 44]. Additionally, CXCL12 promotes neutrophil infiltration to tumors. Moreover, CXCL12 is a potent attractant of dendritic cells (DCs); CCL21 recruits DCs and regulatory T cells (Tregs) [45]. CCL14 participates in the infiltration of the tumor by anti-cancer TILs. CXCL14 is responsible for immune cell recruitment and maturation and is critical to upregulating major histocompatibility complex class I expression on tumor cells. CCL28 activates CCR10 and causes B cell and T cell migration [46–48].

Epigenetic mechanisms, including DNA methylation, histone posttranslational modifications and chromatin structure regulation, are critical for tumor microenvironment (TME) (including immune cells) interaction. Emerging evidence supports that tumors commonly hijack various methylation mechanisms to escape immune surveillance. Recent studies have identified a strong connection between epigenetics and cytokine production in tumorigenesis [49, 50]. Methyltransferases regulate production of interferons, cytokines and chemokines [51]. KMT3A (SETD2), a methyltransferase, is required for interferon pathway by catalyzing the methylation of STAT1, a key transcription factor of interferon response [52]. DNMT suppresses MHC-I expression on tumor cells [53]. The upregulation of PD-L1 on tumor cells likely results from selection pressure exerted by T cell immune response. Epigenetic mechanisms certainly contribute to upregulation of PDL1 [54]. Methylation regulators KMT6A (EZH2), MBD2, TET2 and demethylase KDM5B have been implicated in lymphocyte development [55]. DNMT3A controls fate decision of early effector CD8 + T cell. Loss of DNMT3A leads to ineffective repression of genes that are supposed to be silenced in effector cells, thus generating fewer effector cells [56]. These studies have revealed special relationship between TME-infiltrating immune cells and DNA methylation modification, beyond RNA degradation. NEFM DNA methylation significantly negatively correlated with various immunomodulators and most chemokines and receptors listed in TISIDB, which also contributed to decreased immune cells infiltration in BRCA.

M1 macrophages are involved in normal Th1 immune responses, whereas M2 macrophages support survival and dissemination of cancer cells via secretion of various factors, including cytokines, chemokines and enzymes, which recruit Tregs intratumorally to suppress antitumor cytotoxicity [57]. We demonstrated that NEFM transcriptional expression was significantly associated with IL-17 signaling pathway and cytokine–cytokine receptor interaction by KEGG enrichment analysis. In breast cancer, we infer that NEFM transcriptional expression may affect survival partially through the decreased M2 macrophage infiltration and anti-tumor cytotoxicity of cytokine–cytokine receptor interaction and IL-17 signaling pathway. However, molecular mechanisms should be further investigated. One potential mechanism by which NEFM methylation associated with poor survival may be NEFM methylation inducing tumor immunosuppression depending on decreased TILs. Other mechanisms underlying relationship of NEFM DNA methylation with immune infiltration and poor prognosis in BRCA may include cytokine–cytokine receptor interaction, viral protein interaction with cytokine and cytokine receptor, chemokine signaling pathway, natural killer cell-mediated cytotoxicity, primary immunodeficiency, T cell receptor signaling pathway, IL-17 signaling pathway and PD-L1 expression and PD-1 checkpoint pathway in cancer, which are significantly associated with NEFM DNA methylation by KEGG enrichment analysis.

It has been reported that interrogation of site-specific CpG sites may be another option for assessing immune infiltration in tumors and may possibly predict response to checkpoint inhibitors [58]. Since NEFM DNA methylation significantly negatively correlated with TILs and many immune pathways (especially PD-L1 expression and PD-1 checkpoint pathway in cancer) in BRCA, the six CpG sites within NEFM promoter associated with poor prognosis may serve as biomarkers for predicting immune infiltration in BRCA. Our results also suggest that demethylation of NEFM might be a strategy to improve the efficiency of immunotherapy. Thus, it is required to further explore the detailed mechanism and function of transcriptional expression/DNA methylation in regulating tumor microenvironment.

## Conclusion

NEFM transcriptional expression positively correlates with favorable prognosis and increased levels of macrophage infiltration in BRCA. After adjusted by tumor purity, NEFM expression correlates with increased infiltration of CD8^+^ T cell, whereas decreased infiltration of B cell. NEFM DNA methylation correlates with poor prognosis and decreased immune infiltration of B cells, CD8 + and CD4 + T cells, macrophages, neutrophils and dendritic cells in BRCA. Moreover, NEFM expression/DNA methylation correlates with diverse immune markers and pathways in BRCA. Therefore, our study highlights potential clinical significance of NEFM transcriptional expression/DNA methylation in breast cancer and provides insight into a novel role of NEFM expression/DNA methylation in tumor immune infiltration.

## Methods

### BRCA data and sources

TCGA BRCA DNA methylation profiles (Infinium HumanMethylation450K and HumanMethylation27K) of 1226 breast tissues (884 HumanMethylation450K and 342 HumanMethylation27K samples) and gene expression profiles (IlluminaHiSeq_RNA-SeqV2) of 1223 breast tissues as well as clinical were downloaded from TCGA data repository (https://tcga-data.nci.nih.gov/tcga/). Then, data of immune infiltrates in BRCA were downloaded from TIMER database (http://timer.cistrome.org/).

### Expression levels of NEFM in various types of cancer

The expression profiling of NEFM in various types of cancer was identified in the Oncomine database (https://www.oncomine.org/resource/login.html) [26]. The threshold was determined according to the following values: *p*-value of 0.001, fold change of 1.5, and gene ranking of all. We also analyzed NEFM expression in different types of cancer in TIMER database.

### Prognosis assessment

The Kaplan–Meier plotter and univariable Cox proportional hazards regression models were used to estimate association between NEFM transcriptional expression or DNA methylation and median survival time. Multivariable Cox proportional hazards regression models were used to evaluate impacts of NEFM expression on OS in the presence of other known risk factors. NEFM associated with OS and RFS of BRCA patients was validated in Kaplan–Meier Plotter database (http://kmplot.com/analysis/) [27] among 5,143 BRCA patients. Potential effects of NEFM expression on OS were evaluated in Pan-cancer RNA-seq in Kaplan–Meier plotter database. The association of NEFM protein expression with BRCA OS was analyzed by The Human Protein Atlas database (http://www.proteinatlas.org/).

### Immune infiltration

The data of immune infiltration in BRCA were downloaded from TIMER database. Relationship of NEFM transcriptional expression or DNA methylation with the abundance of immune infiltration was evaluated, including B cells, CD4 + T cells, CD8 + T cells, neutrophils, macrophages, M1 macrophages, M2 macrophages and dendritic cells by R package. We also analyzed the relationship of NEFM expression with the abundance of immune infiltration using gene modules in TIMER. Gene expression level normalized with tumor purity was displayed on the leftmost panel.

There were 28 TIL elements in TISIDB database. Relationship of NEFM transcriptional expression/DNA methylation with abundance of TILs (including activated CD8^+^ T cell, activated CD4^+^ T cell, activated dendritic cell, activated B cell, macrophage, and neutrophil) was examined in TISIDB database.

In addition, the relationship of NEFM transcriptional expression or DNA methylation with immune gene markers was explored via Spearman’s correlation. These immune gene markers included immunomodulators collected from Charoentong's study [28], chemokines and receptors based on TISIDB database [29]. The correlation scatter plots between NEFM transcriptional expression/DNA methylation and immune infiltration levels of immune cells, in BRCA, together with Spearman’s correlation and estimated statistical significance, were described. The log2RSEM value of NEFM expression and log2β value of NEFM DNA methylation were used for x-axis, whereas related immune infiltration levels of immune cells for y-axis. Specific levels of gene markers were displayed with log2RSEM.

TISIDB (http://cis.hku.hk/TISIDB/index.php) is a web portal for tumor and immune system interaction, which integrates multiple heterogeneous data sets. The relative abundance of TILs as demonstrated by 28-gene immune-related signature from Charoentong's study was estimated by using gene set variation analysis (GSVA) based on gene expression profile in TISIDB database (Additional file 3) [29].

TIMER [30] is a comprehensive resource for systematic analysis of immune infiltrates across diverse cancer types (https://cistrome.shinyapps.io/timer/). TIMER applies a deconvolution, a previously published statistical method, to infer relative abundance of tumor-infiltrating immune cells from gene expression profiles. TIMER database includes 10,897 samples across 32 cancer types from The Cancer Genome Atlas (TCGA) to estimate relative abundance of immune infiltration.

### Enrichment analysis

Differentially expressed genes associated with NEFM expression and levels of NEFM DNA methylation were analyzed with DESeq2 R package. Volcano plots and heatmaps were presented. KEGG (Kyoto Encyclopedia of Genes and Genomes) enrichment analyses were performed with R package to identify pathways related to NEFM transcriptional expression/NEFM DNA methylation. A *p*-value of < 0.05 was considered as statistically.

### Statistical analyses

Overall survival (OS) was calculated from the date of diagnosis to death due to any causes or to last follow-up. Recurrence-free survival (RFS) was calculated from the date of diagnosis to local relapse/recurrence or regional relapse/recurrence or death (all causes) whichever occurs first. The Kaplan–Meier method and log-rank test were used to estimate the relationship of NEFM transcriptional expression/DNA methylation with OS and RFS. The Fisher exact and Wilcoxon rank-sum tests were used, respectively, for categorical and continuous variables, to assess the relationship of NEFM expression levels and clinical or molecular characteristics. Multivariable Cox proportional hazards regression models were used to evaluate potential impact of NEFM expression on OS in the presence of other known risk factors. Student's t-test and multiple hypothesis correction (false discovery rate, FDR) were used to identify differences in genome-wide genes, methylation profiles between NEFM^high^ and NEFM^low^ groups. Spearman correlation analysis was performed to evaluate the relationship of NEFM methylation with transcriptional expression or other genes. A *p* value of less than 0.05 was considered statistically significant. All analyses were performed using R 3.6.1 software packages.

## Supplementary Information

**Additional file 1.** The differentially expressed genes between NEFM high-expression group and NEFM low-expression group.

**Additional file 2.** The differentially expressed genes between NEFM high-methylation group and NEFM low-methylation group.

**Additional file 3.** 28 tumor-infiltrating lymphocytes (TILs) and related gene signatures in TISIDE database.

## Data Availability

All data generated or analyzed during this study are included in this published article and its supplementary information files.

## Abbreviations

NEFM: Neurofilament medium
BRCA: Breast cancer
NEFH: Neurofilament heavy
NEFL: Neurofilament light
oAECs: Ovine amniotic epithelium
MDD: Major depressive disorder
LOH: Loss of heterozygosity
DRIP: Dopamine receptor-interacting protein
OS: Overall survival
DFS: Recurrence-free survival
DNMT1: DNA methyltransferase 1
DNMT3A: DNA methyltransferase 3 alpha
DNMT3B: DNA methyltransferase 3 beta
ADORA2A: Adenosine A2a receptor
CSF1R: Colony-stimulating factor 1 receptor
IDO1: Indoleamine 2,3-dioxygenase 1
KDR: Kinase insert domain receptor
LAG3: Lymphocyte-activating 3
TGFB1: Transforming growth factor beta 1
TGFBR1: Transforming growth factor beta receptor 1
VTCN1: V-set domain containing T cell activation inhibitor 1
VSIR (C10ORF54): V-set immunoregulatory receptor
CD276: CD276 molecule
CD40: CD40 molecule
CD70: CD70 molecule
ENTPD1 (CD39): Ectonucleoside triphosphate diphosphohydrolase 1
NT5E (CD73): 5'-Nucleotidase ecto
PVR (CD155): PVR cell adhesion molecule
TMEM173: Transmembrane protein 173
TNFRSF13B: TNF receptor superfamily member 13B
TNFRSF17: TNF receptor superfamily member 17
TNFSF4: TNF superfamily member 4
CXCL12: C-X-C motif chemokine ligand 12
TGFB1: Transforming growth factor beta 1
HTN1: Histatin 1
MAGEA10: MAGE family member A10
CRISPLD2: Cysteine-rich secretory protein LCCL domain containing 2
HTR7: 5-Hydroxytryptamine receptor 7
FST: Follistatin
ASPN: Aspirin
FLRT2: Fibronectin leucine-rich transmembrane protein 2
CPXM1: Carboxypeptidase X, M14 family member 1
SCN4B: Sodium voltage-gated channel beta subunit 4
PTN: Pleiotrophin
PAK5: P21 (RAC1)-activated kinase 5
CCR4: C–C motif chemokine receptor 4
CD96: CD96 molecule
CD226: CD226 molecule
TIGIT: T cell immunoreceptor with Ig and ITIM domains
TAM: Tumor-associated macrophages
Tregs: Regulatory T cells
TILs: Tumor-infiltrating lymphocytes

## References

[1] Esteva F, Hubbard-Lucey V, Tang J, Pusztai L (2019). Immunotherapy and targeted therapy combinations in metastatic breast cancer. Lancet Oncol.

[2] Jeschke J, Bizet M, Desmedt C, Calonne E, Dedeurwaerder S, Garaud S (2017). DNA methylation-based immune response signature improves patient diagnosis in multiple cancers. J Clin Invest.

[3] Ignatiadis M, Singhal S, Desmedt C, Haibe-Kains B, Criscitiello C, Andre F (2012). Gene modules and response to neoadjuvant chemotherapy in breast cancer subtypes: a pooled analysis. J Clin Oncol..

[4] Adams S, Gatti-Mays M, Kalinsky K, Korde L, Sharon E, Amiri-Kordestani L (2019). Current landscape of immunotherapy in breast cancer: a review. JAMA Oncol.

[5] Schmid P, Adams S, Rugo H, Schneeweiss A, Barrios C, Iwata H (2018). Atezolizumab and nab-paclitaxel in advanced triple-negative breast cancer. N Engl J Med.

[6] Denkert C, Loibl S, Noske A, Roller M, Müller B, Komor M (2010). Tumor-associated lymphocytes as an independent predictor of response to neoadjuvant chemotherapy in breast cancer. J Clin Oncol.

[7] Wein L, Savas P, Luen S, Virassamy B, Salgado R, Fio Loi SJ (2017). Clinical validity and utility of tumor-infiltrating lymphocytes in routine clinical practice for breast cancer patients: current and future directions. Front Oncol.

[8] Calmon M, Jeschke J, Zhang W, Dhir M, Siebenkäs C, Herrera A (2015). Epigenetic silencing of neurofilament genes promotes an aggressive phenotype in breast cancer. Epigenetics.

[9] Kudo L, Parfenova L, Vi N, Lau K, Pomakian J, Valdmanis P (2010). Integrative gene-tissue microarray-based approach for identification of human disease biomarkers: application to amyotrophic lateral sclerosis. Human Mol Genetics.

[10] Skvortsova V, Shadrina M, Slominsky P, Levitsky G, Kondratieva E, Zherebtsova A (2004). Analysis of heavy neurofilament subunit gene polymorphism in Russian patients with sporadic motor neuron disease (MND). EJHG..

[11] Mersiyanova I, Perepelov A, Polyakov A, Sitnikov V, Dadali E, Oparin R (2000). A new variant of Charcot-Marie-Tooth disease type 2 is probably the result of a mutation in the neurofilament-light gene. Am J Human Genetics.

[12] Bergson C, Levenson R, Goldman-Rakic P, Lidow M (2003). Dopamine receptor-interacting proteins: the Ca(2+) connection in dopamine signaling. Trends Pharmacol Sci.

[13] Strous R, Greenbaum L, Kanyas K, Merbl Y, Horowitz A, Karni O (2007). Association of the dopamine receptor interacting protein gene, NEF3, with early response to antipsychotic medication. Int J Neuropsychopharmacol.

[14] Alholle A, Brini A, Gharanei S, Vaiyapuri S, Arrigoni E, Dallol A (2013). Functional epigenetic approach identifies frequently methylated genes in Ewing sarcoma. Epigenetics.

[15] Huang Z, Zhuo Y, Shen Z, Wang Y, Wang L, Li H (2014). The role of NEFL in cell growth and invasion in head and neck squamous cell carcinoma cell lines. J Oral Pathol Med.

[16] Revill K, Wang T, Lachenmayer A, Kojima K, Harrington A, Li J (2013). Genome-wide methylation analysis and epigenetic unmasking identify tumor suppressor genes in hepatocellular carcinoma. Gastroenterology.

[17] Emi M, Fujiwara Y, Nakajima T, Tsuchiya E, Tsuda H, Hirohashi S (1992). Frequent loss of heterozygosity for loci on chromosome 8p in hepatocellular carcinoma, colorectal cancer, and lung cancer. Cancer Res.

[18] Vogelstein, B,E Fearon, S Kern, S Hamilton, A Preisinger, Y Nakamura,  (1989). Allelotype of colorectal carcinomas. Science.

[19] Yaremko M, Kutza C, Lyzak J, Mick R, Recant W, Westbrook C (1996). Loss of heterozygosity from the short arm of chromosome 8 is associated with invasive behavior in breast cancer. Genes Chromosomes Cancer.

[20] Hagihara A, Miyamoto K, Furuta J, Hiraoka N, Wakazono K, Seki S (2004). Identification of 27 5' CpG islands aberrantly methylated and 13 genes silenced in human pancreatic cancers. Oncogene.

[21] Kim M, Chang X, LeBron C, Nagpal J, Lee J, Huang Y (2010). Neurofilament heavy polypeptide regulates the Akt-beta-catenin pathway in human esophageal squamous cell carcinoma. PLoS ONE.

[22] Dubrowinskaja N, Gebauer K, Peters I, Hennenlotter J, Abbas M, Scherer R (2014). Neurofilament heavy polypeptide CpG island methylation associates with prognosis of renal cell carcinoma and prediction of antivascular endothelial growth factor therapy response. Cancer Med.

[23] Tabarés-Seisdedos R, Rubenstein J (2009). Chromosome 8p as a potential hub for developmental neuropsychiatric disorders: implications for schizophrenia, autism and cancer. Mol Psychiatry.

[24] Barboni B, Russo V, Curini V, Martelli A, Berardinelli P, Mauro A (2014). Gestational stage affects amniotic epithelial cells phenotype, methylation status, immunomodulatory and stemness properties. Stem Cell Rev Rep.

[25] Lucca L, Axisa P, Aloulou M, Perals C, Ramadan A, Rufas P (2016). Myelin oligodendrocyte glycoprotein induces incomplete tolerance of CD4(+) T cells specific for both a myelin and a neuronal self-antigen in mice. Eur J Immunol..

[26] Rhodes D, Kalyana-Sundaram S, Mahavisno V, Varambally R, Yu J, Briggs B (2007). Oncomine 3.0: genes, pathways, and networks in a collection of 18,000 cancer gene expression profiles. Neoplasia.

[27] Györffy B, Lanczky A, Eklund A, Denkert C, Budczies J, Li Q (2010). An online survival analysis tool to rapidly assess the effect of 22,277 genes on breast cancer prognosis using microarray data of 1,809 patients. Breast Cancer Res Treat.

[28] Charoentong P, Finotello F, Angelova M, Mayer C, Efremova M, Rieder D (2017). Pan-cancer immunogenomic analyses reveal genotype-immunophenotype relationships and predictors of response to checkpoint blockade. Cell Rep.

[29] Ru B, Wong C, Tong Y, Zhong J, Zhong S, Wu W (2019). TISIDB: an integrated repository portal for tumor-immune system interactions. Bioinformatics.

[30] Li T, Fan J, Wang B, Traugh N, Chen Q, Liu J (2017). TIMER: a web server for comprehensive analysis of tumor-infiltrating immune cells. Cancer Res.

[31] Chen K, Liu J, Liu S, Xia M, Zhang X, Han D (2017). Methyltransferase SETD2-mediated methylation of STAT1 is critical for interferon antiviral activity. Cell.

[32] Peng G, Yuan X, Yuan J, Liu Q, Dai M, Shen C (2015). miR-25 promotes glioblastoma cell proliferation and invasion by directly targeting NEFL. Mol Cell Biochem.

[33] Ricketts C, Morris M, Gentle D, Shuib S, Brown M, Clarke N (2013). Methylation profiling and evaluation of demethylating therapy in renal cell carcinoma. Clin Epigenetics.

[34] Pasculli B, Barbano R, Parrella P (2018). Epigenetics of breast cancer: biology and clinical implication in the era of precision medicine. Semin Cancer Biol.

[35] Bartos A, Fialová L, Soukupová J, Kukal J, Malbohan I, Pit'ha J (2007). Elevated intrathecal antibodies against the medium neurofilament subunit in multiple sclerosis. J Neurol..

[36] Jones A, Mowry B, McLean D, Mantzioris B, Pender M, Greer J (2014). Elevated levels of autoantibodies targeting the M1 muscarinic acetylcholine receptor and neurofilament medium in sera from subgroups of patients with schizophrenia. J Neuroimmunol.

[37] Lucca L, Desbois S, Ramadan A, Ben-Nun A, Eisenstein M, Carrié N (2014). Bispecificity for myelin and neuronal self-antigens is a common feature of CD4 T cells in C57BL/6 mice. J Immunol.

[38] Zhang S, Wang Y, Gu Y, Zhu J, Ci C, Guo Z (2018). Specific breast cancer prognosis-subtype distinctions based on DNA methylation patterns. Mol Oncol.

[39] Zarour H (2016). Reversing T-cell Dysfunction and Exhaustion in Cancer. Clin Cancer Res.

[40] Allard B, Longhi M, Robson S, Stagg J (2017). The ectonucleotidases CD39 and CD73: novel checkpoint inhibitor targets. Immunol Rev.

[41] Li Y, Zhou Q, Song Q, Wang R, Lyu S, Guan X (2020). Overexpression of an immune checkpoint (CD155) in breast cancer associated with prognostic significance and exhausted tumor-infiltrating lymphocytes: a cohort study. J Immunol Res.

[42] Guo Q, Betts C, Pennock N, Mitchell E, Schedin P (2017). Mammary gland involution provides a unique model to study the TGF-β cancer paradox. J Clin Med.

[43] Do H, Lee C, Cho J (2020). Chemokines and their receptors: multifaceted roles in cancer progression and potential value as cancer prognostic markers. Cancers.

[44] Schmall A, Al-Tamari H, Herold S, Kampschulte M, Weigert A, Wietelmann A (2015). Macrophage and cancer cell cross-talk via CCR2 and CX3CR1 is a fundamental mechanism driving lung cancer. Am J Respir Crit Care Med.

[45] Mollica Poeta V, Massara M, Capucetti A, Bonecchi R (2019). Chemokines and chemokine receptors: new targets for cancer immunotherapy. Front Immunol.

[46] Westrich J, Vermeer D, Colbert P, Spanos W, Pyeon D (2020). The multifarious roles of the chemokine CXCL14 in cancer progression and immune responses. Mol Carcinog.

[47] Korbecki J, Kojder K, Simińska D, Bohatyrewicz R, Gutowska I, Chlubek D (2020). CC chemokines in a tumor: a review of pro-cancer and anti-cancer properties of the ligands of receptors CCR1, CCR2, CCR3, and CCR4. Int J Mol Sci..

[48] Korbecki J, Grochans S, Gutowska I, Barczak K, Baranowska-Bosiacka I (2020). CC chemokines in a tumor: a review of pro-cancer and anti-cancer properties of receptors CCR5, CCR6, CCR7, CCR8, CCR9, and CCR10 ligands. Int J Mol Sci.

[49] McDonald K, Kawaguchi T, Qi Q, Peng X, Asaoka M, Young J (2019). Tumor heterogeneity correlates with less immune response and worse survival in breast cancer patients. Ann Surg Oncol.

[50] Roulois D, Loo Yau H, Singhania R, Wang Y, Danesh A, Shen S (2015). DNA-demethylating agents target colorectal cancer cells by inducing viral mimicry by endogenous transcripts. Cell.

[51] Chiappinelli K, Strissel P, Desrichard A, Li H, Henke C, Akman B (2015). Inhibiting DNA methylation causes an interferon response in cancer via dsRNA including endogenous retroviruses. Cell.

[52] Peng D, Kryczek I, Nagarsheth N, Zhao L, Wei S, Wang W (2015). Epigenetic silencing of TH1-type chemokines shapes tumour immunity and immunotherapy. Nature.

[53] Li H, Chiappinelli K, Guzzetta A, Easwaran H, Yen R, Vatapalli R (2014). Immune regulation by low doses of the DNA methyltransferase inhibitor 5-azacitidine in common human epithelial cancers. Oncotarget.

[54] Zhu H, Bengsch F, Svoronos N, Rutkowski M, Bitler B, Allegrezza M (2016). BET bromodomain inhibition promotes anti-tumor immunity by suppressing PD-L1 expression. Cell Rep.

[55] Cao J, Yan Q (2020). Cancer epigenetics, tumor immunity, and immunotherapy. Trends Cancer.

[56] Ladle B, Li K, Phillips M, Pucsek A, Haile A, Powell J (2016). De novo DNA methylation by DNA methyltransferase 3a controls early effector CD8+ T-cell fate decisions following activation. Proc Natl Acad Scie USA.

[57] Solinas G, Germano G, Mantovani A, Allavena P (2009). Tumor-associated macrophages (TAM) as major players of the cancer-related inflammation. J Leukocyte Biol.

[58] Bacolod M, Barany F, Fisher P (2019). Can CpG methylation serve as surrogate markers for immune infiltration in cancer?. Adv Cancer Res.

